# Long-term effects of the Mediterranean lifestyle program: a randomized clinical trial for postmenopausal women with type 2 diabetes

**DOI:** 10.1186/1479-5868-4-1

**Published:** 2007-01-17

**Authors:** Deborah J Toobert, Russell E Glasgow, Lisa A Strycker, Manuel Barrera, Debra P Ritzwoller, Gerdi Weidner

**Affiliations:** 1 Oregon Research Institute, 1715 Franklin Blvd, Eugene, OR 97403, USA; 2 Kaiser Permanente Colorado, Denver, Colorado 80231, USA; 3 Psychology Department, Arizona State University, Box 871104 Tempe, AZ 85287, USA; 4 Kaiser Permanente Colorado, Clinical Research Unit, 580 Mohawk Dr., Boulder, CO 80302, USA; 5 Preventive Medicine Research Institute, 900 Bridgeway, Sausalito, California 94965, USA

## Abstract

**Background:**

Multiple-risk-factor interventions offer a promising means for addressing the complex interactions between lifestyle behaviors, psychosocial factors, and the social environment. This report examines the long-term effects of a multiple-risk-factor intervention.

**Methods:**

Postmenopausal women (*N *= 279) with type 2 diabetes participated in the Mediterranean Lifestyle Program (MLP), a randomized, comprehensive lifestyle intervention study. The intervention targeted healthful eating, physical activity, stress management, smoking cessation, and social support. Outcomes included lifestyle behaviors (i.e., dietary intake, physical activity, stress management, smoking cessation), psychosocial variables (e.g., social support, problem solving, self-efficacy, depression, quality of life), and cost analyses at baseline, and 6, 12, and 24 months.

**Results:**

MLP participants showed significant 12- and 24-month improvements in all targeted lifestyle behaviors with one exception (there were too few smokers to analyze tobacco use effects), and in psychosocial measures of use of supportive resources, problem solving, self-efficacy, and quality of life.

**Conclusion:**

The MLP was more effective than usual care over 24 months in producing improvements on behavioral and psychosocial outcomes. Directions for future research include replication with other populations.

## Background

Age-adjusted mortality from coronary heart disease (CHD) has been declining in men with and without diabetes, and in women without diabetes, but increasing in women with diabetes [1]. CHD remains the leading cause of death among women in the U.S. [2] and is increasing [3]. Risk of and death from CHD is significantly higher among postmenopausal women and is 2.5 times higher among women with vs. without diabetes [4]. The prevalence of diabetes is increasing [5]. Given that the age-adjusted prevalence of diabetes for women was less than 3% in the 1980s but reached nearly 5% in 2004 [6], there is an urgent need to investigate interventions designed to enhance lifestyle behaviors and reduce the risks of CHD associated with diabetes.

The evidence suggests that among people with type 2 diabetes, diet [7], physical activity [8,9], stress [10], smoking [11], and social resources [12,13] are key modifiable CHD risk factors. Multiple-risk-factor interventions could be powerful methods for addressing the complex interactions between lifestyle behaviors [14] and the physical and social environment [15-17]. However, reports of the sustainability of changes made in these interventions are largely absent from study results [18] and maintenance remains a major challenge [19,14]. Modification of longstanding lifestyle behaviors, such as diet, physical activity, and smoking, appears to be especially difficult. Many individuals engage in new lifestyle behaviors for short periods, but are typically unable to maintain these changes for long [19]. However, evidence is needed from studies with longer follow-ups (3–5 years) that a consistent and positive factor related to maintenance is multiple rather than single behavioral targets [20].

The Mediterranean Lifestyle Program (MLP) is a theory-based comprehensive lifestyle management intervention designed to reduce CHD risk in postmenopausal women with type 2 diabetes. The MLP was adapted from successful programs for middle-aged persons with CHD [21] and for women with CHD [22,23]. In the first 6 months of the MLP, significant improvement was found in multiple behaviors, as previously reported [24,25]. This paper evaluates the longer-term effects of the MLP. The main hypothesis was that those randomized to the MLP, compared to a randomized usual care (UC) condition, would show greater 12- and 24-month improvements in targeted lifestyle behaviors, including eating patterns, physical activity, and stress management, and in psychosocial variables, including perceived social support, problem-solving, self-efficacy, and quality of life.

The RE-AIM framework was used to plan and evaluate the MLP's public health impact based on program Reach, Effectiveness, Adoption, Implementation, and Maintenance. The RE-AIM framework was developed in part to encourage greater emphasis on issues of external validity and thus help close the gap between research and adoption, a gap that can be attributed at least partially to the almost exclusive preoccupation of health promotion research with efficacy studies focused on internal validity. The RE-AIM model encompasses the dimensions of Reach (participant participation rate and representativeness); Effectiveness (including impact on quality of life and potential negative outcomes); Adoption (by representative settings and clinicians); Implementation consistency by various staff; and Maintenance at both the patient and the setting levels.

## Methods

### Study design

Full details of the study design have been published elsewhere [24,25]. A sample of 250 study participants was determined to yield 90% power (α = .05, two-tailed) to detect a moderate effect (*f *= .30) for the primary dependent variables of dietary and physical activity outcomes, allowing for 20% attrition. A total of 279 postmenopausal women with type 2 diabetes, who were patients of participating primary care clinics, were recruited for the study. Inclusion criteria were: having type 2 diabetes for at least 6 months, being postmenopausal, living independently, having a telephone, ability to read English, not being developmentally disabled, and living within 30 miles of the intervention site (Eugene, OR, USA). Exclusion criteria included being older than 75 years of age or planning to move from the area within the study's time span. All patients meeting eligibility criteria were sent letters from their primary care providers, followed by phone calls inviting them to participate. Fifty-one percent of eligible women contacted agreed to participate in the study. Enrollees were representative of patients in participating primary care offices and the diabetes population of the state. A detailed description of the recruitment procedures, the adoption of the program by physicians, and its reach among patients is presented elsewhere [26]. Participants were stratified prior to randomization on physician practice, smoking status, and type of diabetes medication.

The research protocol was approved by the Oregon Research Institute Institutional Review Board (FWA 00005934), and written informed consent was obtained from all study participants prior to participation. All activities involving human subjects were in accordance with the Declaration of Helsinki, the Belmont Report, and U.S. regulations governing the protection of human research subjects. The study participants received no monetary compensation for completing assessments.

### Intervention

#### MLP condition

The conceptual basis for this program, detailed in a previous publication [27], combined Social Cognitive Theory [28], Goal Systems [29], and Social Ecological Theory [30]. This model has evolved to include multiple system and social-environmental factors, including social support, that influence self-management of chronic illness [30]. Participants were able to set personal lifestyle change goals at the start of the intervention, and then received ongoing peer and professional support for their goals throughout the treatment program.

The MLP was delivered by a registered dietitian, an exercise physiologist, a stress-management instructor, and a combination of professional and lay support group leaders. The program started with a 2 1/2-day non-residential retreat, which was followed by 4-hour weekly meetings consisting of 1 hour each of Mediterranean-style potluck, physical activity, stress management, and support groups. The intervention was conducted in four sequential waves, 2 months apart, with approximately 40 treatment condition and 30 control condition participants in each wave. This schedule was necessary to keep the group size manageable within staff and space constraints.

The first 6 months of the intervention were designed to teach the program components and build group cohesion.

##### Mediterranean diet

The project registered dietitian taught participants the Mediterranean alpha-linolenic acid-rich diet, which is low in saturated fat but moderately high in more healthful monounsaturated fats [31]. Individualized carbohydrate and fat recommendations were provided to optimize blood glucose and lipid concentrations. The Mediterranean diet recommended more bread; more root vegetables, green vegetables, and legumes; more fish; less red meat (e.g., beef, lamb, pork), replaced by poultry; daily fruit; and avoidance of butter and cream, to be replaced by olive/canola oil or olive/canola-based margarine. MLP participants were asked to complete and bring to some weekly meetings a simple self-monitoring log of their adherence to the Mediterranean diet components.

##### Physical activity

The initial physical activity goal, developed in consultation with the project's exercise physiologist, was consistent with the recent Centers for Disease Control and Prevention and the American College of Sports Medicine guidelines for physical activity [32]: 30 minutes of moderate physical activity on most days of the week. Once that goal was achieved, participants were advised to build up to 1 hour of moderate aerobic activity daily. Women who had engaged in little or no activity before the program were helped to set individualized goals to gradually increase activity, by adding about 5 minutes per session or more days per week of exercise.

##### Stress management

Using procedures from Ornish [21] and Toobert et al. [23], participants were instructed in yoga, progressive deep relaxation, meditation, and directed or receptive imagery. The purpose of each technique was to increase the sense of relaxation, concentration, and awareness. Participants were asked to practice all of these techniques for at least 1 hour per day and received a videotape to assist them.

A variety of motivational techniques were employed to keep the meetings interesting and to boost attendance, including contests, self-monitoring, and group and individual rewards.

After 6 months of intervention, MLP participants were further randomized to one of two maintenance conditions: (1) a faded schedule of weekly meetings led by lay leaders or (2) four meetings over 18 months with project staff to complete a personalized, computer-assisted program. This personalized support condition was designed to enhance use of social and environmental resources for healthful lifestyle changes. An interactive computer-based program was developed to assess an individual's use of available supportive resources, and to help users set goals for taking better advantage of these resources. These two maintenance conditions were set up to test whether targeted, computer-assisted enhancement of social support promoted better maintenance of initial gains than continued group weekly meetings. The two maintenance conditions were compared separately to the control condition at 12 and 24 months. With about 80 study participants in each maintenance condition and 116 in the control group, sufficient power existed to detect an effect size of *f *= .30.

##### Usual care control condition

Participants in the control condition completed all assessment procedures. These participants received no additional intervention beyond usual care from their physicians.

### Measures

Women were assessed in groups of 6–8 at Oregon Research Institute in Eugene, OR. Some demographic measures were collected on the telephone for screening purposes prior to randomization; all other measures were collected at baseline prior to randomization and at 6, 12, and 24 months following introduction of the lifestyle program.

To ensure adherence to the assessment schedule, a variety of techniques were used. Ten days prior to the assessment, participants were mailed pre-visit packets which included a reminder letter with the date of their visit, a description of the assessment and what to bring, and two surveys to complete. Transportation needs were met by the project, if necessary, usually by providing a taxi and occasionally by arranging carpooling. Though rare, child care and/or elder care costs also were covered when requested. Assessments were scheduled so that friends could attend the same session. The assessors created a welcoming environment so that many participants looked forward to their assessment visits. Also used to boost assessment participation were telephone reminders, holiday and birthday cards, continued address updates, and flexibility (a 3-month window) in the time frame for assessment completion.

A relatively large number of measures was required because of the many anticipated effects of the multiple-risk-factor intervention, the need to measure each of the multiple behavioral targets, and the lack of gold standards for measuring most of the behaviors. Multivariate analyses of variance were used to limit experiment-wide error and to provide a stronger, more robust approach than the arbitrary selection of one behavioral or process measure.

#### Behavioral outcomes

##### Dietary

The semi-quantitative food frequency questionnaire (FFQ) developed at the Fred Hutchinson Cancer Research Center [33] was used to document percent of calories from saturated fat. This FFQ has been validated with 4-day food records and 24-hour dietary recalls (average correlation *r *= .5). The validity of the percent of dietary saturated fat measured by the FFQ was assessed in this study by saturated fatty acids from a plasma fatty acid profile. Using the control group only, the 24-month correlation between intake of saturated fat measured by the FFQ and plasma concentrations of saturated fatty acids was *r *= .26, *p *< .01.

##### Physical activity

The CHAMPS Activities Questionnaire for Older Adults [34] provided an estimate of kilocalories/kilogram/hour of moderate-intensity exercise-related activities, which incorporates the three key components of physical activity: frequency, duration, and intensity. The CHAMPS is a widely used measure that has been shown to be sensitive to change in similar populations. Women also were monitored for 7 days with the Yamax DW-500 pedometer to record the number of steps taken daily. Pearson product-moment correlation coefficients between the CHAMPS scale and the pedometer recordings were averaged for the four study time points, yielding a moderate but significant result (*r *= .28, *p *< .001).

##### Stress management

**Figure 1:**
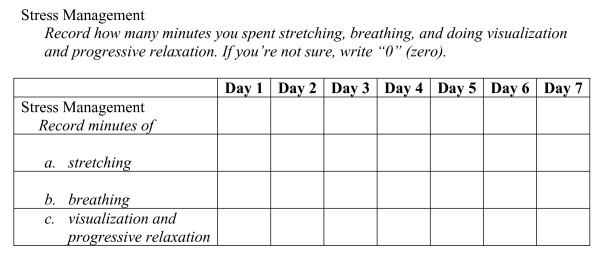
7-Day Self-Monitoring Chart.

Since objective measures of stress-management practices are not well-established, a self-monitoring form was designed for this study (Figure 1). Participants monitored their daily performance of at least 20 minutes of yoga stretches, 5 minutes of breathing exercises, 15 minutes of progressive relaxation, and 5 minutes each of meditation and visualization. Each scale consisted of the number of self-monitored minutes of stretching, breathing, or meditation/visualization for each of 7 days. Each of the three subscales was made up of 7 items or days. Trunk flexion and shoulder range-of-motion tests were conducted to assess changes in flexibility resulting from the yoga practices.

##### Social desirability

To evaluate and adjust for the relations between social desirability and the behavioral and psychosocial measures, the Balanced Inventory of Desirable Responding [35] was used. Paulhus [36] reported a coefficient alpha of .83 for the all items, and test-retest correlations of between .65 and .69.

### Psychosocial measures

#### Social resources

Since the MLP intervention targeted different sources of support (e.g., friends, family, health care providers), measures were included to address each of these components. The Brief Chronic Illness Resources Survey (CIRS) [30] provided a profile of an individual's support for behavior-specific disease management, ranging from more proximal support (e.g., family and friends) to more distal factors (e.g., neighbourhood or community). In these analyses, the total score from the CIRS was used to represent received support. The UCLA [37] measures three types of support (i.e., informational, tangible, emotional), three dimensions of support (i.e., amount, satisfaction, reciprocity), and four sources of support (i.e., friends, relatives, partner, organizations). For this project, two other sources of support – people in a support group and medical care providers – were added to the original UCLA. The Total Positive and Total Negative scales were included in these analyses.

#### Problem-solving ability

The Diabetes Problem-Solving Interview was developed for adults with type 2 diabetes [38]. For this study, the interview was modified to ask respondents to write a description of how they would react to nine scenarios presenting potential challenges to program adherence. Coders scored responses to produce an average rating of problem-solving skill. Inter-rater reliability on these scores ranged from *r *= .57 to *r *= .90 and averaged *r *= .72. Six-month test-retest reliabilities using only the control group was *r *= .51 for overall skill ratings.

#### Self-efficacy

Two measures of self-efficacy were used to assess different areas of diet and physical activity-related obstacles. Confidence in Overcoming Challenges to Self-Care was used to assess confidence overcoming obstacles to adhering to diet, physical activity, and stress-management [39]. This 49-item instrument assesses confidence in overcoming such factors as cost, time, social pressures, competing demands, and thoughts associated with achieving one's dietary, physical activity, and stress-management goals. Recent analyses of this instrument [39] demonstrated reasonable psychometric properties for a brief scale (test-retest reliability *r *= .60; internal consistency (*α *= .50). The total score was used in these analyses. Participants' self-efficacy for achieving their dietary and physical activity goals was assessed using the Sallis Self-Efficacy for Diet and Exercise Behavior instrument [40]. This instrument assesses confidence in performing diet and exercise behaviors for at least 6 months. The Eating Behavior scale contains 20 items (test-retest reliability ranged from *r *= .43 to .64; Cronbach's α .84) and the Self-Efficacy for Exercise scale contains 12 items (test-retest reliability was *r *= .68; α coefficients ranged from .85 to .93) [40].

#### Depression

Since CHD and diabetes are closely linked to depression [13]., the Center for Epidemiologic Studies Depression Scale (CES-D) was administered. The CES-D is a general measure of depressive symptoms that has been shown to have good reliability and validity [41], and has been used extensively in epidemiologic studies.

#### Perceived stress

The Perceived Stress Scale was administered to determine whether perceived stress changed in conjunction with the stress-management intervention. This 14-item measure, based on the transactional stress theory of Lazarus, has been found to be an independent and significant predictor of physical symptoms and health behaviors after controlling for psychological symptoms [42].

#### Quality of life

The Diabetes Distress Scale (DDS), a diabetes-specific measure of quality of life, also was used. Respondents rated the degree to which common barriers to adherence were problematic for them (lower ratings indicate better perception of quality of life). This scale assessed diabetes-specific overall emotional distress, interpersonal distress, regimen-related distress, and physician-related distress. A recent 32-item revision of the DDS [43] produces subscales on these four dimensions as well as an overall score; lower scores indicate better quality of life. The DDS has good internal reliability with the four subscales (*α *= 0.87), and has been shown to be responsive to psychosocial intervention [43]. Two of the four DDS scales were used: the regimen-related and interpersonal distress scales.

#### Cost analysis

Using methods described in detail elsewhere [44], an economic analysis of the costs associated with the MLP intervention was conducted. The analysis was conducted primarily from the perspective of the potential adopting organization or potential payer, such as Medicare or a health insurance plan. The analysis evaluated the cost of delivering the MLP compared to the UC condition. Using retrospective data collected during the development and implementation of the MLP, estimates were made of total intervention costs, incremental costs associated with the intervention group relative to the comparison condition, costs per participant, and marginal costs per incremental improvement in several of the primary outcomes. Total intervention costs were estimated as the sum of the costs associated with recruitment of participants (in the intervention arm) and staff; labor costs associated with the time spent by educators, dietitians, physicians, exercise physiologists, meeting leaders, and support group leaders; retreats; training, phone charges, and supplies; and rent attributable to facility space needed for group meetings. All assessment costs were excluded.

### Statistical methods

Descriptive analyses, including means, standard deviations, and distributions, were used to clean the data, determine whether transformations were needed, and describe overall level of improvement and implementation. One-way analyses of variance (ANOVA) were used to evaluate between-condition differences at baseline. Repeated measures multivariate analyses of covariance (MANCOVA), covarying out the effect of baseline scores, were used to compare long-term results on each of the three sets of outcome measures at 6, 12, and 24 months across conditions. Follow-up univariate analyses of covariance (ANCOVA) were conducted to reveal the source of effects only when the MANCOVA was significant in a given domain. To aid interpretation, the results reported in all tables have not been adjusted for baseline values.

#### Additional covariates

Prior to conducting the repeated measures MANCOVAs, univariate statistics and correlation matrices were examined for potential additional correlates of the behavioral and psychosocial dependent variables. For the behavioral outcomes, social desirability, income, number of comorbid conditions, and age were all entered as covariates, and none was a significant contributor to the models. Social desirability was entered as a covariate in all of the psychosocial analyses presented.

#### Missing data

All analyses were performed two ways. First, a complete-case approach was used. Participants with missing follow-up data on the outcome variable of interest were excluded from the analysis. Second, identical analyses were conducted after missing data were imputed using EMCOV [45]. Significance and conclusions from imputed analyses were mostly identical or stronger compared to complete-case analyses. For ease of understanding, the nonimputed results are presented in the tables, with the imputed results, where different, described in the text.

## Results

### Study participants

#### Usual care vs. MLP

Women randomized to the MLP intervention were similar to women in the UC condition. The baseline characteristics of women assigned to UC vs. MLP have been reported in Toobert et al. (2003) [24] and are presented for convenience in Table 1. As can be seen, this was a high-risk group of women: Their average BMI exceeded 35 kg/m^2 ^and most had multiple chronic illnesses.

**Table 1:** Characteristics of participants by treatment condition

	Mean (SD) or Percent		
Characteristic	Usual Care (N = 116)	MLP (*N *= 163)	Significance (*p *Value)

Age	60.7 (7.8)	61.1 (8.0)	.70
Weight (kg)	93.9 (23.8)	92.3 (21.2)	.55
Waist/hip ratio	0.9 (.08)	0.91 (.08)	.35
Body mass index (kg/m^2^)	35.6 (8.8)	35.1 (7.7)	.62
Age diagnosed with diabetes	52.5 (10.7)	53.0 (10.0)	.71
Years taking medications	5.0 (6.3)	4.9 (4.9)	.90
Years diagnosed with diabetes	8.5 (8.3)	8.2 (7.3)	.77
Current smoker (% yes)	10.3	8.7	.59
Income			.22
% $0 to $ 9,999	14.9	5.8	
% $10,000 to $19,999	21.9	24.5	
% $20,000 to $29.999	17.5	23.9	
% $30,000 to $39,000	17.5	14.2	
% $40,000 to $49,000	10.5	11.0	
% $50,000 to $59,999	9.5	5.8	
% $60,000 to $69,999	0.9	5.2	
% $70,000 to $79,999	1.7	3.9	
% $80,000 or more	5.2	5.8	
Type of glucose-lowering medication			.62
% None	17.2	24.7	
% Oral medication only	61.2	54.9	
% Insulin only	12.1	7.4	
% Insulin and oral medication	9.5	13.0	
Present living arrangement			.43
% With spouse	49.1	51.5	
% With spouse and children	11.2	9.8	
% With children or others	9.4	14.7	
% Alone	30.2	23.9	
Level of education achieved			.72
% 0 to 11^th ^grade	11.2	8.5	
% High school graduate	25.0	25.2	
% Some college	39.7	43.6	
% College/university graduate	24.1	22.7	
% Caucasian	94.8	92.0	.59
Medications			
% Taking lipid-lowering	40.5	38.9	.79
% Taking blood pressure-lowering	47.4	46.3	.86
% Taking estrogen replacement therapy	46.6	59.3	.04
Number of comorbidities			.27
% With no other disease	4.3	4.9	
% With 1–2 other diseases	43.1	50.9	
% With ≥ 3 other diseases	52.6	44.2	
Most prevalent comorbidities			
% Having CHD	15.0	14.0	.26
% Having arthritis	50.9	56.4	.36
% Having high blood pressure	72.4	70.6	.74
% Having back problems	37.9	33.1	.41

#### Participants vs. dropouts

Twenty-four-month follow-up data were collected for 237 (85%) of the randomized participants. Analyses of the characteristics of dropouts vs. participants present at 24-month follow-up revealed no significant main effects or interactions with treatment condition on baseline characteristics of age, weight, waist/hip ratio, body mass index, smoking status, type of medication, income, education, living arrangement, diabetes complications, or number of comorbidities. Significant differences were found as follows: Those who remained in the study at 24 months were older at diabetes diagnosis (mean age of diabetes diagnosis for dropouts was 49.6 vs. 53.4 years for those who remained in the study), and had taken medications for less time (mean years taking diabetes medications for dropouts was 7.3 vs. 4.5 years for those who remained in the study). There was also a significant difference in employment status between those retained and those who dropped out at 24 months. Specifically, 34.3% (*N *= 81) of those who remained in the study were employed compared to 55.8% (*N *= 24) of dropouts.

#### Intraclass correlation coefficients (ICCs)

ICCs were computed for key dependent variables to determine whether there was significant clustering by wave of the study participants. All ICCs were less than .003 (median = .001), indicating an absence of wave effects.

#### Implementation (Attendance)

Of 23 meetings offered over the first 6 months, the mean (SD) number of sessions attended for the MLP participants was 12.4 (5.7); the range was 0–20. Of 39 meetings offered from 6–24 months for the 63 weekly meeting participants, the mean (SD) number of sessions attended was 19.4 (13.2), or 50%; the range was 0–38. Of the four sessions offered from 6–24 months for the 82 personalized computer-assisted condition participants, the mean (SD) number of sessions attended was 2.5 (1.8), or 63%; the range was 0–4.

#### Maintenance between-condition effects

Analyses revealed few significant and no meaningful differences between the two experimental maintenance conditions; given the small sample size, these two conditions were merged and compared to the UC condition in all further analyses.

### Behavioral outcomes

Results are presented on the following behavioral measures or sets of measures: (1) dietary behavior, (2) physical activity, and (3) stress management. Since each of the three overall repeated measures MANCOVAs was significant, univariate analyses were conducted to reveal the source of the effects. Behavioral results are summarized in Table 2.

**Table 2:** Behavioral outcomes for usual care (UC) and intervention (MLP) conditions at baseline, and 6, 12, and 24 months

	Baseline Mean (SD)	6 months Mean (SD)	12 months Mean (SD)	24 months Mean (SD)	Between subjects *F*	Condition by time *F*
Diet (% calories saturated fat)						
UC	.13 (.04)	.12 (.04)	.12 (.04)	.12 (.04)	39.30***	2.74
MLP	.14 (.03)	.10 (.03)	.11 (.03)	.11 (.03)		
Physical Activity (kcal/kg/hr moderate activity)						
UC	7.72 (8.44)	7.56 (6.66)	6.39 (6.73)	7.13 (5.29)	7.62 **	.11
MLP	5.63 (5.30)	8.78 (6.51)	7.69 (6.35)	7.90 (6.55)		
Stress Management						
Daily Practice (minutes)						
UC	10.3 (19.8)	11.7 (19.4)	9.4 (20.5)	11.0 (19.2)	20.48***	0.94
MLP	6.1 (14.0)	20.8 (17.4)	16.0 (16.5)	16.8 (16.4)		
Flexibility (sit-and-reach % score)						
UC	32.9 (25.7)	31.0 (27.2)	28.6 (28.3)	30.4 (28.4)	3.99*	0.32
MLP	32.3 (23.9)	36.2 (27.3)	32.2 (28.6)	36.2 (29.0)		
Flexibility (range-of-motion % score)						
UC	10.8 (21.2)	9.5 (19.6)	6.4 (16.7)	9.8 (22.4)		
MLP	14.7 (24.1)	15.2 (24.9)	14.7 (25.1)	14.4 (25.9)		

*Note*: Baseline values, social desirability, income, age, and comorbidities were entered as covariates. Based on repeated measures MANCOVAs (and follow-up ANOVAs) using complete cases (*N *= 118 for CHAMPS; *N *= 192 for all other analyses). Means are not adjusted for baseline levels or covariates. * = *p *< .05; ** = *p *< .01; *** = *p *< .001.

#### Dietary patterns

MLP participants showed significantly greater improvement overall, at all follow-up assessment points, on percent of calories from saturated fat than did the UC group. FFQ data indicated that the intervention produced significant 24-month between-condition effects and consistent improvements in mean percent calories from saturated fat (average reduction in percent calories from saturated fat was 3% for MLP vs. 1% for UC).

#### Physical activity

Physical activity improvements also were maintained over 24 months. A significant between-condition effect was found on the repeated measures ANCOVA for the kcals/kg/hr moderate-intensity activity measure derived from the CHAMPS, showing the MLP conditions to be superior to the UC condition in frequency, duration, and intensity of physical activity.

#### Stress management

Stress-management results from the 7-day self-monitoring log indicated that MLP participants significantly increased the number of daily minutes they practiced stress-management techniques (yoga stretches, breathing, guided visualization, and meditation) compared to UC participants, and these effects maintained across all time points. The imputation analysis yielded similar results with the exception that the condition-by-time effect became significant.

There was a significant between-condition effect for the flexibility repeated measures MANCOVA, which included both the sit-and-reach and range-of-motion tests. For the sit-and-reach test, flexibility significantly improved in the MLP group and worsened in the UC condition. Range-of-motion flexibility was greater at each time point in the MLP compared to the UC; however both conditions decreased their range-of-motion flexibility over the course of the study.

### Psychosocial outcomes

#### Social resources

Repeated measures MANCOVAs revealed a significant improvement in perceived social support (see Table 3), *F*(3,195) = 13.68, *p *< .001. Follow-up ANCOVAs indicated improvements in the total perceived Positive and Negative Support scales measured by the UCLA Social Support Inventory. Imputation analyses revealed an additional condition-by-time effect for the UCLA Positive scale and a loss of the significant between-group effect for the UCLA Negative scale. The total score of the brief CIRS also showed a significant long-term treatment effect in favor of the MLP participants on use of community and social environmental resources.

**Table 3:** Psychosocial outcomes for usual care (*UC*) and intervention (*MLP*) conditions at baseline, and 6, 12, and 24 months

	Baseline Mean (SD)	6 months Mean (SD)	12 months Mean (SD)	24 months Mean (SD)	Between subjects *F*	Condition by time *F*
Social Support						
UCLA Negative Summary Score						
UC	3.95 (.46)	3.99 (.53)	4.03 (.47)	3.99 (.53)	3.98*	1.48
MLP	3.98 (.45)	3.91 (.54)	3.92 (.53)	3.99 (.53)		
UCLA Positive Summary Score						
UC	2.95 (.60)	2.99 (.71)	3.00 (.77)	2.99 (.71)	30.11***	2.96^a^
MLP	2.85 (.73)	3.40 (.74)	3.28 (.74)	3.20 (.79)		
Chronic Illness Resources Survey						
UC	2.77 (.55)	2.77 (.59)	2.77 (.60)	2.78 (.58)	5.76*	1.47
MLP	2.69 (.58)	2.92 (.65)	2.84 (.52)	2.80 (.56)		
Self-Efficacy						
Confidence to Overcome Challenges						
UC	3.1 (.73)	3.0 (.79)	3.0 (.78)	2.9 (.76)	11.43***	.47
MLP	3.0 (.77)	3.1 (.73)	3.1 (.77)	3.1 (.81)		
Sallis Self-Efficacy for Diet and Exercise						
UC	4.1 (.58)	3.8 (.71)	3.9 (.71)	3.8 (.66)	14.51***	1.33
MLP	4.1 (.59)	4.1 (.54)	4.0 (.59)	4.1 (.62)		
Quality of Problem-Solving Strategies						
UC	3.6 (.68)	3.6 (.78)	3.9 (.70)	3.8 (.68)	11.73***	1.35
MLP	3.6 (.77)	3.9 (.77)	4.1 (.67)	4.0 (.72)		
Depression (CES-D)						
UC	15 (10)	15 (12)	14 (9)	14 (10)	0.34	2.33
MLP	12 (11)	13 (11)	15 (11)	12 (11)		
Perceived Stress (Cohen)						
UC	2.7 (.57)	2.6 (.59)	2.6 (.58)	2.6 (.61)	0.90	3.70*
MLP	2.6 (.63)	2.5 (.62)	2.6 (.66)	2.4 (.64)		
Self-Monitored Stress						
UC	1.59 (.43)	1.60 (.47)	1.59 (.49)	1.58 (.44)	1.59	.36
MLP	1.58 (.38)	1.52 (.36)	1.55 (.47)	1.52 (.44)		
Quality of Life (DDS)						
Regimen-Related Scale						
UC	17.9 (5.8)	15.7 (5.2)	15.5 (5.6)	15.2 (5.2)	3.45^a^	1.03
MLP	18.7 (5.3)	14.8 (4.7)	15.1 (4.6)	15.0 (4.9)		
Interpersonal Distress Scale						
UC	12.5 (5.0)	11.2 (4.4)	11.2 (4.5)	10.6 (3.7)	1.13	.21
MLP	12.8 (4.9)	11.7 (4.4)	11.5 (4.9)	11.2 (4.6)		

*Note*. Baseline values and social desirability were entered as covariates. Based on repeated measures MANCOVAs (and follow-up ANOVAs) using complete cases (*N*s ranged from 194 for Self-monitored stress to 204 for most of the other measures). Means are not adjusted for baseline levels or covariates. * = *p *< .05; ** = *p *< .01; *** = *p *< .001; ^a ^= *p *< .1.

#### Problem-solving ability

A significant overall effect was found on the repeated measures ANCOVA for problem-solving skills. The effect was due primarily to significantly larger improvements among the MLP participants on the quality of the problem-solving strategies employed.

#### Self-efficacy

MLP participants made and maintained modest but significantly greater improvements than UC participants on measures of dietary self-efficacy, exercise self-efficacy, and confidence in overcoming challenges to illness management.

#### Stress and depression

As shown in Table 3, the separate repeated measures MANCOVAs revealed only one significant effect for perceived stress and no significant effects for depression or self-monitored stress.

#### Quality of life

As shown in Table 3, despite a pattern of greater improvement for the MLP condition on the regimen-related subscale, the overall repeated measures MANCOVA for the quality of life (DDS) regimen-related and interpersonal distress scales did not reach the *p *< .05 level of significance for the MLP compared to UC.

#### Cost

Total direct and indirect intervention costs were estimated to be $409,165, or $2,510 per MLP participant relative to UC for the 24-month period ($309,302 direct costs). Using the same data employed to estimate results in Table 2, change scores were calculated for measures demonstrating statistically significant improvements. For the behavioral improvements, there was a cost of $221 per unit improvement in calories derived from saturated fat as measured by the FFQ, $1,434 per kilocalorie increase in physical activity, and $1,168 per unit improvement in daily stress management.

## Discussion

The MLP produced substantial and broad benefits. Both the magnitude and the maintenance of the behavioral effects on dietary and physical activity outcomes were impressive, as were the stress-management results. The psychosocial and quality-of-life differences favouring MLP over UC were generally consistent and maintained well over the 24-month follow-up period, but were not as large. From a RE-AIM perspective, the MLP program produced strong Reach, Adoption, Effectiveness, and Maintenance results. The costs of the intervention were moderately high and Implementation (attendance) was modest, suggesting targets for future improvement.

It is noteworthy that the MLP produced enhancements rather than decrements in quality of life, an important finding with a program requiring a significant time commitment. Especially encouraging were the between-condition effects on multiple process measures of social and community support, problem-solving ability, and self-efficacy, as these psychosocial processes have been independently shown to be major predictors of long-term behavior change [28,46,47].

The breadth of improvements was striking. The MLP produced consistent and significantly greater improvements than the UC in all of the four diverse CHD behavioral risk factors targeted – eating patterns, physical activity, social support, and stress management. There were too few smokers to analyze tobacco use effects. It is difficult to produce lasting improvements in single behavioral risk factors [48], and the demonstration of sustained effects on both multiple behaviors and potential underlying processes is encouraging.

This study has both methodological strengths and limitations. A strength of the study is use of the RE-AIM framework and inclusion of cost and cost-effectiveness measures. Both the cost and the cost-effectiveness estimates are relatively high compared to other estimates, and there are likely multiple reasons for this. The most obvious is that this was an intensive intervention that continued for 24 months and the costs of ongoing group meetings added up to a large sum over time. It could be that alternative or less-expensive maintenance approaches could be developed. The women who participated in this trial were at extremely high risk for further CHD-related diseases, which are major causes of health care expenditures as well as mortality. For them, a program with this level of intensity may be warranted, as the overall cost of the intervention is considerably less than invasive surgical or even some intensive pharmacologic or cardiac rehabilitation interventions [45]. Second, an earlier cost analysis on this project at 6 months revealed considerably lower cost and cost-effectiveness results [44]. The higher estimates at follow-up are partially due to shrinking differences between intervention and control conditions decreased over time, caused by some slippage in the intervention condition and improvements on some outcomes (e.g., quality of life) in the control condition. Finally, the cost measures, which included recruitment, an expensive interactive CD-ROM program, and indirect costs, were more comprehensive than those in many other estimates that have been limited to time directly spent in intervention.

Other strengths include the generally representative sample of a high-risk group of female participants, the randomized design, the breadth of measures employed, the relatively high reach and participation rates for an intensive intervention, the targeting of multiple risk behaviors, high adoption rates by primary care providers throughout the community (70%), and the consistency of results.

Additional research is needed to determine whether these improvements translate into even longer-term reductions of health-related costs. Although the sample was heterogeneous on demographic factors such as income, the group was relatively ethnically and racially homogenous, consisting primarily of white women. Future replications with diverse populations are indicated to further evaluate the promise and translation potential of the MLP.

The extended length of follow-up and ability to retain 85% of this high-risk, older sample are also strengths. The study adds to growing evidence of the importance of exploring alternative methods for achieving long-term outcomes.

Related to its potential for translation into practice, the MLP performed well on the RE-AIM dimensions of Reach, Effectiveness, Adoption, Implementation, and Maintenance [49]. Thus, judging by the RE-AIM evaluation framework, this line of research has considerable public health potential, given the high risk for CHD and complications among women with diabetes and the long-term patterns of reductions in behavioral risk factors for CHD [50]. The only RE-AIM criterion related to potential for translation [51] not evaluated to date is longer-term maintenance of effects without further intervention. The question of longer-term maintenance is being addressed in a continuation of this study, which will provide 3- to 7-year maintenance data and will lay the groundwork for translating a successful lifestyle change intervention into practice.

Although a surprising number of high-risk women were willing to participate in this project, cost-conscious health care systems may not be. It is interesting to note, however, that, given the high costs of treating cardiovascular disease, Centers for Medicare and Medicaid Services is conducting demonstration projects on lifestyle interventions that are even more intensive than the MLP. Landmark behavior change/self-management adherence studies in diabetes – namely the Diabetes Control and Complications Trial (DCCT) [52] and the Diabetes Prevention Program [53] – were both relatively intense. It may be that intensive and continued intervention is necessary to produce lasting changes in lifestyle behaviors within the "obesogenic" environment in which we live [54].

## Conclusion

Long-term maintenance of changes made in lifestyle interventions, especially multiple-risk- factor interventions, are largely absent from study results [18], and maintenance is a major challenge [19,14]. In this study, participants randomized to the MLP, compared to a usual care (UC) control condition, showed greater long-term (12- and 24-month) improvements in multiple lifestyle behaviors, including eating patterns, physical activity, and stress management as well as psychosocial outcomes. Additional research is needed to determine whether these improvements could be translated into further reductions of health-related costs and with diverse populations.

## Abbreviations

MLP: Mediterranean Lifestyle Program; CHD: coronary heart disease; UC: usual care; FFQ: food frequency questionnaire; CHAMPS: Community Healthy Activities Model Program for Seniors; CIRS: Brief Chronic Illness Resources Survey; CES-D: Center for Epidemiologic Studies Depression Scale; DDS: Diabetes Distress Scale; ANOVA: one-way analyses of variance; MANCOVA: multivariate analyses of covariance; EMCOV: estimation of means and covariances; UCLA: University of California, Los Angeles; ENRICHD: Enhancing Recovery in CHD Patients; RE-AIM: Reach, Effectiveness, Adoption, Implementation, and Maintenance; DCCI: Diabetes Control and Complications Trial.

## Competing interests

The author(s) declare that they have no competing interests.

## Authors' contributions

DJT conceived of the study, and directed all aspects of the study, including development, assessment, and analyses, and led the writing of the manuscript. REG, MB Jr., LAS, and GW contributed to the development of assessment and intervention materials, recruitment procedures, and assessment and intervention protocols, and assisted with the writing of the manuscript.
